# Lifecourse transitions, gender and drinking in later life

**DOI:** 10.1017/S0144686X15001178

**Published:** 2015-10-26

**Authors:** CLARE HOLDSWORTH, MARTIN FRISHER, MARINA MENDONÇA, CESAR DE OLIVEIRIA, HYNEK PIKHART, NICOLA SHELTON

**Affiliations:** * School of Physical and Geographical Sciences, Keele University, UK.; † School of Pharmacy, Keele University, UK.; ‡ Centre for Social Policy, Keele University, UK.; § Department of Epidemiology and Public Health, University College London, UK.

**Keywords:** alcohol consumption, older adults, gender, partnership, health, lifecourse

## Abstract

Older people consume less alcohol than any other adult age group. However, in recent years survey data on alcohol consumption in the United Kingdom have shown that while younger age groups have experienced a decline in alcohol consumption, drinking behaviours among the elderly have not reduced in the same way. This paper uses data from the English Longitudinal Study of Ageing to analyse both the frequency and quantity of older adult's alcohol consumption using a lifecourse approach over a ten-year period. Overall drinking declined over time and the analysis examined how socio-economic characteristics, partnership, employment and health statuses were associated with differences in drinking behaviours and how these changed over time. Higher wealth and level of education were associated with drinking more and drinking more frequently for men and women. Poorer self-rated health was associated with less frequent consumption and older people with poor and deteriorating health reported a steeper decline in the frequency of alcohol consumption over time. Men who were not in a partnership drank more than other men. For women, loss of a partner was associated with a steeper decline in drinking behaviours. These findings have implications for programmes to promote responsible drinking among older adults as they suggest that, for the most part, characteristics associated with sustaining wellbeing in later life are also linked to consuming more alcohol.

## Introduction

Older adults' alcohol consumption has come under increasing popular and academic scrutiny in recent years. In the United Kingdom (UK), for example, recent media coverage has drawn attention to the ‘hidden’ reality of alcohol misuse among older adults (*see e.g.* Bingham 2014; McVeigh 2013). While reports of alcohol misuse among the elderly point out that the majority of older adults are moderate drinkers, popular interest in drinking practices in later life demonstrates the need for better understanding of this behaviour. Moreover, the unveiling of older adults' drinking behaviours reveals normative assumptions about alcohol and age, that drinking – particularly when associated with loss of control – is a behaviour characteristic of youth and older adults are expected to internalise responsibility (*see e.g.* Griffin *et al*. 2009; Johnson 2010). Yet drinking is an important dimension of older adults' lives, for many it is a way of connecting with others, as well as with their past lives (Burruss, Sacco and Smith 2014; Ward, Barnes and Gahagan 2011). This paper responds to this public concern about drinking in later life through exploring how drinking behaviours in later life vary according to key socio-economic and lifecourse characteristics and how these behaviours change over a ten-year period. The analysis uses a lifecourse perspective, which considers how drinking behaviours are structured by trajectories and transitions within social contexts (Schulenberg, Maggs and O'Malley 2003). The analysis uses data from the English Longitudinal Study of Ageing (ELSA) to investigate how socio-economic characteristics, partnership, employment and health statuses influence drinking behaviours among ELSA participants at the beginning of the survey, and how lifecourse transitions relating to health, employment and partnership are associated with changes in drinking behaviours over the ten-year period. The analyses are stratified by gender as gender identity shapes both lifecourse trajectories and transitions (Rossi 1985) and drinking behaviours (Wilsnack *et al*. 2009). By comparing men's and women's drinking over the lifecourse, we can contextualise the relationships between lifecourse events and alcohol consumption. The paper begins with an overview of existing research on drinking in later life to identify the main trends in drinking over the lifecourse and characteristics that have informed our analysis. We then present the data-set and the modelling strategy used. The empirical analysis investigates both the frequency and quantity of older adults' drinking to provide a more detailed understanding of how these two attributes of drinking behaviours change in later life. In particular, the analysis considers the importance of lifecourse transitions in accelerating or decelerating changes in alcohol consumption.

## Trends in alcohol consumption and health in later life

One important observation about the relationship between age and alcohol consumption is that there is no universal pattern of consumption trends with age that is consistent across time and place (Wilsnack *et al*. 2009). However, the prevailing pattern in Anglo-European contexts is that alcohol consumption declines in later life (Molander, Yonker and Krahn 2010; Platt, Sloan and Costanzo 2010; Shaw *et al*. 2011) after peaking in young adulthood (Meng *et al*. 2014); and more older adults are non-drinkers compared to younger age groups (Meng *et al*. 2014; Ng Fat and Fulller 2012). This overall decline in consumption with age is found for all consumption levels, including heavy drinking (Karlamangla *et al*. 2006). However, while older adults are more likely to be non-drinkers and consume less if they do drink, they are also more likely to drink more frequently; in contrast to younger people who are more inclined to concentrate consumption in binge-drinking episodes (Ng Fat and Fuller 2012). English data on the frequency of drinking show that the regularity of drinking increases with age (Meier 2010). Men in the oldest age group (75 and over) were more likely to drink five or more times a week than any other age group, and women aged 65–74 were the most frequent drinkers (Meier 2010).

The overall decline in alcohol consumption with age has meant that national alcohol misuse prevention strategies do not identify older adults' drinking as a priority for intervention (*see e.g.* the UK's 2012 Alcohol Strategy; Secretary of State for the Home Office 2012). Yet this policy and research lacuna has been addressed in recent years. This has been driven by a number of factors. First, in a global ageing society the number of older drinkers is increasing (International Center for Alcohol Policies 2014). Second, and a more fundamental concern, is that the age profile of drinking is changing along with trends in per capita consumption. This change is brought about by age, period and cohort effects, and disentangling these is not necessarily straightforward (Meng *et al*. 2014). However, what is emerging from analysis of cross-sectional data is that there have been greater discernible changes in younger people's drinking compared to older age groups in recent years. For example, in the UK there was a substantial increase in per capita consumption during the latter half of the twentieth century, particularly among women (Smith and Foxcroft 2009), which has been reversed slightly in the first decade of the twenty-first century (Meng *et al*. 2014). This recent decline in drinking has been concentrated in younger age groups (Meier 2010) as British young people born since 1980 are not reproducing the equivalent high consumption/low abstinence behaviours of earlier cohorts (Meng *et al*. 2014). However, their predecessors who had higher consumption and lower abstinence during youth and mid-adulthood are now entering later life. While these cohorts might experience the age effect of declining consumption/increasing abstinence, the overall impact of these age and cohort trends is that differentials in drinking across the lifecourse have declined in recent years (Office for National Statistics 2013). In the UK, men aged 45–64 are more likely to drink above recommended limits than all other ages, though there is no age effect of heavy drinking for women (Meier 2010).

Turning to the health outcomes of drinking, data on alcohol-related mortality and morbidity also highlight a potential upward trend associated with drinking in later life. Alcohol-related mortality is higher among older age groups and is increasing among the elderly while stabilising and declining at younger ages (Knott, Scholes and Shelton 2013; Office for National Statistics 2013). Alcohol-related morbidity for older adults is also increasing. Between 2002 and 2010 in England and Wales, the number of alcohol-related admissions into hospital for men aged 65 and over increased by 175 per cent and for women by 145 per cent (Institute for Alcohol Studies 2013). Increases in alcohol-related mortality and morbidity in later life will not just reflect changes in drinking among the elderly, but will also be due to longer-term health effects of drinking across the lifecourse (Bergmann *et al*. 2013). However, relative increases in the number of older adults drinking in excess of recommended levels and incidences of old-age alcohol mortality and morbidity have focused policy attention on the need to respond to older person's drinking (Crome *et al*. 2011).

## Characteristics of drinking in later life

Research on drinking in later life has sought to identify factors associated with the age profile of drinking to understand better changing consumption over the lifecourse, particularly the overall decline in drinking among older adults (Brennan *et al*. 2011; Platt, Sloan and Costanzo 2010). Understanding the causality of why older adults consume less, if not less often, and the characteristics and lifecourse events that may accelerate or decelerate this decline, can inform estimates of the drinking behaviours of current generations entering later life (Shaw *et al*. 2011). Factors that are associated with drinking in later life include health status, socio-demographic characteristics and lifecourse transitions, which all point to the significance of the social context of drinking (Platt, Sloan and Costanzo 2010).

Health is one of the most important factors associated with drinking at all ages, yet the causality between health and alcohol consumption is complex. There is considerable policy and research interest in how different levels of alcohol consumption impact on health and wellbeing and in discerning both the harms and possible benefits of drinking (Balsa *et al*. 2008; Chen and Hardy 2009; Holahan *et al*. 2014; Hsu *et al*. 2014). Epidemiological analysis has considered the potential benefits of moderate alcohol consumption for health and cognitive function in later life (*see e.g.* Hagger-Johnson *et al*. 2013; Hogenkamp *et al*. 2014; Holahan *et al*. 2010; Kim *et al*. 2012; Lang *et al*. 2007; Lin, Guerrieri and Moore 2011). A distinctive characteristic of drinking at all ages is that poor health is associated with abstinence as part of a J or U-shape relationship between alcohol consumption and health; that is, poor health outcomes are associated with adults who either abstain from alcohol or are heavy drinkers (Gunzerath *et al*. 2004; Knott *et al*. 2015; Ng Fat 2012; Polen *et al*. 2010). At one end of the drinking spectrum, life-time heavy alcohol consumption has been shown to increase the risk of dying, especially from cardiovascular disease (Bergmann *et al*. 2013). However, the association between poor health and abstinence is more debated. It may be explained by the ‘sick quitter’ effect; that is, individuals with poor health stop drinking or reduce their alcohol consumption (Brennan, Schutte and Moos 2010*a*; Shaper 2011; Shaper, Wannamethee and Walker 1988). Alternatively, it could occur because of health benefits associated with moderate drinking, as moderate drinkers report better health outcomes particularly for cardiovascular disease (Ronksley *et al*. 2011) One of the challenges in unravelling this relationship is that it is difficult to control fully for the ‘sick quitter’ effect in survey analysis and the causal explanation for this behaviour is unclear. Disentangling the causality between health and drinking is particular problematic in later life, as cross-sectional survey analysis does not find an association between heavy drinking and poor health at older ages (Frisher *et al*. 2015). This could be due to a selection effect associated with the increased risk of dying for heavy consumers, resulting in an under-representation of older heavy drinkers with poor health. In older ages the relationship between poor health and less consumption/abstinence is intensified as health declines with age (Dawson, Goldstein and Grant 2013). Cessation of drinking associated with poor health could be due to abstinence associated with previous as well as existing poor health; medical advice about reducing alcohol consumption in response to a health condition; possible interactions with medication; and, it also might reflect limited social connections for people living with poor health who as a consequence have fewer opportunities to drink (Ng Fat *et al*. 2014). In summary, the relationship between health status and drinking is not straightforward and this appears to be intensified in later life. It is necessary to consider multiple causalities between alcohol consumption and health, as not only does drinking impact on health, but health status can also influence drinking behaviours.

Other factors associated with declining alcohol consumption with old age point to the importance of individual resources and identities in mediating the relationship between drinking and age. Gender is an important determinant of drinking behaviours at all ages across the lifecourse. Women drink less, less often and are more likely to abstain compared to men (French *et al*. 2014). In recent years this differential has declined, particularly for younger age groups (Alati *et al*. 2014), and increases in younger women's consumption have been an important drive in increases in overall per capita consumption (Smith and Foxcroft 2009). At older ages the differential by gender remains and in quantitative analysis is often one of the most important variables in predicting drinking behaviours, including problem drinking (Moos *et al*. 2009). Studies of women's drinking behaviours have sought to situate these in the context of identity development and performance, and as such emphasise the conditional rather than essentialist nature of these behaviours (Day, Gough and McFadden 2004). This also raises the possibility that gendered practices will vary with age, though the intersection between gender and age over the lifecourse has received less consideration.

Gender also intersects with socio-economic characteristics in framing drinking behaviours across the lifecourse and in later life (Platt, Sloan and Costanzo 2010). Individual resources are an important moderating factor for consumption levels, with an overall positive relationship between income and consumption occurring in different national contexts (Brennan, Schutte and Moos 2010*b*). Recent analysis of ELSA data demonstrates that characteristics associated with ‘successful ageing’ are also associated with increased risk in harmful drinking (Iparraguirre 2015). However, the precise relationship between social disadvantage and alcohol consumption is not necessarily linear. Studies that focus on deprivation have found a U-shape relationship as more disadvantaged individuals are more likely to abstain or misuse alcohol drinking (Cerda, Johnson-Lawrence and Galea 2011). At older ages this U-shape is less marked, and the evidence points to a closer association between disadvantage and abstinence.

It is not just individual resources and identities that influence drinking behaviours over the lifecourse. Drinking is, in most contexts, a social behaviour and one that is conditional on relationships with others over time and place, though the social context of drinking has been explored mostly with reference to younger people's drinking (Kuntsche *et al*. 2005). Yet it is reasonable to assume that changes in older adults' drinking behaviours may be brought about by lifecourse transitions that impact on social connectivity. Dealing with isolation and loneliness is an important challenge in promoting active ageing and may also be associated with increased reliance on alcohol at older ages (Wadd *et al*. 2011). Qualitative research with older adults has identified social isolation as a causal factor of drinking; in particular, widowhood is a recognised risk factor (Wilson *et al*. 2013). This is partially confirmed in quantitative analysis where not being married is associated with greater alcohol consumption in older age, including binge and heavy drinking (Blazer and Wu 2009; Platt, Sloan and Costanzo 2010). Moreover, across the lifecourse the impact of marriage on men and women's drinking patterns has also been demonstrated (Demers, Bisson and Palluy 1999; Windle and Windle 2014). However, the dynamics of partnership change on drinking behaviours have received less consideration in existing studies.

Another aspect of the social context of drinking that undergoes change in later life relates to working practices. Organisational cultures have been shown to be relevant in shaping employee's drinking behaviours (Ames, Grube and Moore 2000). Moreover, employment can provide social connections that facilitate consumption, people to go drinking with as well as opportunities to drink. Therefore, it is reasonable to assume that retirement will impact on drinking behaviours. However, this association does not seem to be straightforward as there is no consistent pattern in existing research (Brennan, Schutte and Moos 2010*b*; Kuerbis and Sacco 2012; Wang, Steier and Gallo 2014). While retirement might reduce drinking opportunities for some workers, for employees working in industries with alcohol controls retirement might increase opportunities to drink, and retirees might also have more time to drink. Furthermore, individual experiences of retirement transitions will not be independent of other characteristics, particularly income, as well as changes in health (Brennan, Schutte and Moos 2010*b*). Retirement has very different meanings for older people, and these differences moderate the association with drinking behaviours (Kuerbis and Sacco 2012).

To summarise, existing research on drinking in later life has established the general overall decline in consumption in later life and has identified a number of factors associated with this behavioural change, particularly health. The review of existing research on drinking in later life demonstrates the importance of health, partnership and employment statuses for drinking, though the specific impact of transitions in these statuses on drinking behaviours remains unclear, as research has not to date considered the dynamics between lifecourse transitions in later life, such as changes in partnership status, health and retirement. We propose that a lifecourse perspective which considers the relevance of trajectories and transitions in key life events may be applied to drinking in later life. This approach considers the influence of socio-economic characteristics and partnership and health statuses on drinking behaviours, the trajectories of drinking behaviours over time and how these might be influenced by lifecourse transitions. Our analysis examines how trajectories of drinking in later life are changed by transitions in these three domains (health, employment and partnership), and how this differs by gender.

## Methodology

### Data

The longitudinal analysis of drinking in late life was carried out using ELSA (Steptoe *et al*. 2013). The original ELSA sample was recruited from participants in the Health Survey for England (HSE) for the years 1998, 1999 and 2001, and the responses to the HSE are included in the ELSA study as wave 0. The first wave of ELSA was carried out in 2002/03 with a sample size of 12,099 participants. The sample has been interviewed every two years and the last wave was surveyed in 2012/13. While ELSA has included questions on alcohol consumption in all waves, these have not been consistent in each wave and for this reason we have had to restrict our analysis of drinking behaviours to waves 0 (1998/99/01), 4 (2008/09) and 5 (2010/11). Wave 0 data do not contain the detailed socio-demographic indicators collected in the main ELSA surveys and for the baseline measures of wealth we have used a wave 1 variable. The analysis was restricted to respondents in all three waves (4,738 cases) and we excluded respondents who have either left the study over time or have recruited to refresh it over the ten years. Respondents from all three waves were used as the multi-level models require at least three repeated measures for most cases (Curran, Obeidat and Losardo 2011). Analysis of attrition of key variables has shown that attrition was biased towards non-drinkers rather than drinkers and there was no discernible relationship between being present in all three waves and drinking behaviours for drinkers in wave 0 (Nazroo, Zaninotto and Gjonça 2008).

### Variables

We use two variables to capture drinking behaviours: frequency of drinking in the last 12 months and the number of weekly units consumed.^1^ We model frequency and quantity separately as they are both important determinants of drinking behaviour that may diverge in later life (Ng Fat and Fuller 2012). In particular, analysis of cross-sectional data has found that older adults consume less alcohol but drink more frequently compared to younger adults. Our analysis has considered baseline determinants and trajectories of both frequency and quantity of consumption to provide a more detailed picture of changes in drinking behaviours in later life.

The analysis included various measures of socio-economic characteristics, lifecourse transition and other health behaviours, and the distribution of these variables is given in the Appendix. These include three lifecourse transition variables for partnership, employment status and health. There is considerable complexity in these transitions and in order to include these variables in the longitudinal model with sufficient cases for each transition status we have simplified these as follows. For partnership, we distinguish respondents who remained in a partnership over the study period, those who were not in a partnership in all waves and those who either formed or left a partnership between waves 0 and 5. One of the limitations of this variable is that we have not distinguished between the reasons for ending a partnership due to the small numbers involved (this could be widowhood or divorce/separation). Employment transitions focus on transitions into retirement and distinguish those who remained economically active from those who were always retired or who retired during the study period. Self-rated health was used as an indicator of health as it is a strong predictor of mortality (Jylha 2009; Lima-Costa *et al*. 2012). Transitions in health distinguish between individuals who had constant good or not-good self-rated health (*i.e.* fair or poor health) and transitions between these two. For all the transition variables, more complex transitions that were not captured by the main categories were coded as ‘other’. These residual transitions accounted for approximately 2 per cent of partnership and 3 per cent of health transitions and, due to the small numbers, are not reported here. However, for employment, other transitions accounted for approximately 10 and 30 per cent of male and female respondents, respectively, and this category is reported. All other socio-economic variables were captured at wave 0 (wave 1 for wealth) and include wealth quintiles, level of education and smoking behaviours. In the analysis we also considered variables for social support and social capital (as measured by membership of organisations) but these were not significant and we have not included these in the final models. Age at wave 0 was also included to control for age affects across the sample. We also checked if the relationship with age was linear by including age squared in the analysis, this was not significant and we have not included this variable in the final model.

### Modelling strategies

In our analysis we modelled how frequency and consumption of alcohol varied over the ten-year period for men and women. We have used two modelling strategies to analyse frequency and quantity of alcohol consumption because of the different nature of these outcome variables. Frequency of drinking was captured using a variable on drinking in last 12 months which was a categorical ordered variable, and for this analysis we performed multi-level ordered logit analysis. The analysis of consumption used a measure of the number of units consumed in the previous week which is a continuous variable, and this analysis was carried out using a growth curve model of log of weekly units. We transformed the units of alcohol consumed in the last week into a logarithm because the distribution of units of alcohol was not normally distributed. The longitudinal ELSA data can be viewed as having a two-level hierarchical structure (Singer and Willet 2003). Level 1 described within-person change; that is, how respondents' frequency and quantity of alcohol consumption changed over time. Level 2 described between-person differences in drinking behaviours over time. Data on frequency and consumption were available at three time-points (waves 0, 4 and 5) and the measure of time in the analysis was wave which was fitted as a continuous variable. In this analysis, we investigated how the rate of change over time in frequency and consumption of alcohol varied according to lifecourse transitions. Time trend analysis was included by fitting an interaction term between transitions variables and wave. The analysis therefore provided information about the individuals' initial drinking behaviour (intercept) and trajectory during the study period (slope).

Both types of multi-level analyses were estimated with Stata 13. Analyses were carried out separately by gender as existing research on drinking at all ages identified the significance of gender differences in drinking. The separate analysis by gender considered whether the impacts of both socio-economic characteristics and lifecourse events on drinking behaviours were similar for men and women. The models for frequency of drinking were carried out with all respondents (drinkers and non-drinkers) who were present in waves 0, 4 and 5 (2,046 men and 2,692 women). Non-drinkers were not included in the analysis of weekly consumption as it was not possible to include a value of zero in the dependent variable. Thus, analysis of quantity of consumption was restricted to respondents who were drinkers in at least one wave (1,774 men and 1,889 women).

The models were built in a step-wise fashion to compare the impact of lifecourse events in more detail. The stepwise models were built up as follows: Model 1 included age and wave variables only; Model 2 included age, wave and partnership (including an interaction between partnership and wave); Model 3 included age, wave and employment (including an interaction between employment and wave); Model 4 included age, wave and health (including an interaction between health and wave); and Model 5 (final model) with all lifecourse transition variables, interactions between transition variables, and wave and other socio-economic characteristics included as covariates.

## Results

The distribution of the frequency of drinking in last 12 months and mean weekly units consumed in waves 0 and 5 by gender are given in Tables 1 and 2. Men's greater and more frequent consumption is confirmed, as is the overall decline in quantity and frequency over time for both genders. There were though some notable trends beyond this overall pattern. For frequency, there was a marked increase in abstinence in wave 5 compared to wave 0 for both men and women. For women in wave 5, not drinking was the modal consumption frequency. There were small increases in the proportion of men and women reporting occasional drinking between the two time-points. This increase in abstinence was concomitant with a decline in more frequent drinking, and most of this was accounted for by a reduction in daily drinking, particularly for women. In Table 2, the mean reduction in mean weekly units consumed was roughly equivalent for both men and women (around 28 per cent), though it was slightly greater for men.

**Table 1.** Frequency of drinking by wave and gender

Frequency of drinking	Wave 0		Wave 5	
	Men	Women	Men	Women
N^1^	2,047	2,692	2,016	2,635
	*Percentages*			
Does not drink	6.0	10.8	17.6	26.0
Once/twice a year	3.7	9.6	5.4	12.4
Once every couple of months	4.3	6.7	4.2	7.7
One to two times a month	8.4	14.1	9.4	11.3
One to two days a week	25.6	24.5	23.7	18.6
Three to four days a week	18.6	12.6	13.5	8.5
Five to six days a week	6.3	4.0	5.7	3.6
Almost every day	27.4	17.8	20.4	11.8

*Notes*: Base: English Longitudinal Study of Ageing members in waves 0 and 5. 1. The reduction in the N value between waves 0 and 5 is accounted for by missing data in wave 5, that is respondents did not complete the question on frequency of drinking in wave 5.

**Table 2.** Mean weekly units by gender and wave

	Wave 0	Wave 5
Men	18.9 (1,678)	13.5 (1,332)
Women	9.3 (1,582)	6.7 (1,241)

*Notes*: The number of drinkers in each wave is given in parentheses. Base: English Longitudinal Study of Ageing members who are drinkers in waves 0 and 5.

### Frequency of drinking

The analysis of men's and women's frequency of drinking at baseline (wave 0) and over time (between waves 0, 4 and 5) is summarised in Tables 3 and 4. Taking the baseline coefficients first (*i.e.* variables fitted without an interaction with wave), negative coefficients for a category indicate that individuals with this characteristic drank less frequently than the reference category at wave 0, positive coefficients indicate more frequent drinking. The coefficients for the interactions between the transition variables and wave can be interpreted to indicate whether the rate of change in frequency of drinking over time varies in comparison to the reference category (in a partnership at all waves, in continual employment or in continual good health). A negative coefficient for the interaction terms indicates a steeper decline in frequency over time for a particular sub-category in comparison to the reference group. In other words, as wave is negative in all models, a negative interaction term indicates that respondents in the sub-category in question experienced a steeper decline in frequency of drinking over the ten-year period, compared to the reference category. A positive coefficient suggests that individuals in the particular sub-category experienced a smaller decline in drinking frequency over time.

**Table 3.** Multi-level ordered logit model of drinking frequency: men

Variable	Model 1	Model 2	Model 3	Model 4	Model 5
Wave^1^	−0.254**	−0.225**	−0.213**	−0.154**	−0.141**
Age at wave 0	−0.039**	−0.035**	−0.064**	−0.028**	−0.036**
Partnership (Ref. In partnership all waves):					
Out of partnership all waves		−0.149			0.383*
Enters partnership between waves		0.734			0.724
Partnership ends between waves		−0.145			0.023
Partnership × wave:					
Out of partnership all waves		−0.158**			−0.113**
Enters partnership between waves		0.061			−0.007
Partnership ends between waves		−0.035			−0.002
Employment (Ref. Employed at all waves):					
Retired at all waves			0.877**		0.418
Retires between waves			0.056		−0.054
Other transitions			0.382		0.327
Employment × wave:					
Retired at all waves			−0.107**		−0.048
Retires during study period			0.043		0.069*
Other transitions			−0.164**		−0.067
Self-rated health (Ref. Good health all waves):					
Poor health all waves				−1.321**	−0.730**
Health deteriorates between waves				−0.680**	−0.337
Health improves between waves				−1.288**	−0.942**
Self-rated health × wave:					
Poor health all waves				−0.227**	−0.206**
Health deteriorates between waves				−0.153**	−0.137**
Health improves between waves				−0.094**	−0.090*
Wealth (Ref. Bottom quintile):					
Second quintile					0.721**
Third quintile					0.885**
Fourth quintile					1.028**
Top quintile					1.907**
Education (Ref. No education):					
Compulsory education					0.342*
Post-compulsory education					0.487**
Degree or higher					1.776**
Smoking (Ref. Non-smoker):					
Used to smoke occasionally					−0.026
Used to smoke regularly					1.213**
Current smoker					0.799**

*Notes*: English Longitudinal Study of Ageing participants in all three waves; N = 2,046. 1. In Models 2–5 the coefficient for wave is for the reference group only for partnership (Model 2), employment (Model 3), health (Model 4) and for all transitions variables (Model 5). Ref.: reference category.

*Significance levels*: * *p* < 0.1, ** *p* < 0.05.

**Table 4.** Multi-level ordered logit model of drinking frequency: women

Variable	Model 1	Model 2	Model 3	Model 4	Model 5
Wave^1^	−0.358**	−0.281**	−0.324**	−0.252**	−0.218**
Age at wave 0	−0.062**	−0.043**	−0.077**	−0.043**	−0.029**
Partnership (Ref. In partnership all waves):					
Out of partnership all waves		−0.655**			−0.039
Enters partnership between waves		0.586			0.694
Partnership ends between waves		−0.564**			−0.130
Partnership × wave:					
Out of partnership all waves		−0.152**			−0.113**
Enters partnership between waves		−0.054			−0.110
Partnership ends between waves		−0.150**			−0.119**
Employment (Ref. Employed at all waves):					
Retired at all waves			0.714**		0.567*
Retires between waves			0.007		0.085
Other transitions			0.481*		0.463*
Employment × wave:					
Retired at all waves			−0.059		0.028
Retires during study period			0.046		0.063
Other transitions			−0.118**		−0.037*
Self-rated health (Ref. Good health all waves):					
Poor health all waves				−2.156**	−1.537**
Health deteriorates between waves				−0.449*	−0.124
Health improves between waves				−1.479**	−1.043**
Self-rated health × wave:					
Poor health all waves				−0.188**	−0.165**
Health deteriorates between waves				−0.224**	−0.200**
Health improves between waves				−0.056	−0.037
Wealth (Ref. Bottom quintile):					
Second quintile					0.731**
Third quintile					1.087**
Fourth quintile					1.960**
Top quintile					2.677**
Education (Ref. No education):					
Compulsory education					0.823**
Post-compulsory education					1.150**
Degree or higher					1.665**
Smoking (Ref. Non-smoker):					
Used to smoke occasionally					0.668**
Used to smoke regularly					1.325**
Current smoker					1.016**

*Notes*: English Longitudinal Study of Ageing participants in all three waves; N = 2,692. 1. In Models 2–5 the coefficient for wave is for the reference group only for partnership (Model 2), employment (Model 3), health (Model 4) and for all transitions variables (Model 5). Ref.: reference category.

*Significance levels*: * *p* < 0.1, ** *p* < 0.05.

The coefficients for wave and age in Model 1 demonstrate that over time and at older ages the frequency of drinking declined. The coefficient for wave was greater for women, indicating that over time women's frequency of alcohol consumption declined faster than men's. There were also important differences by gender and between the different lifecourse transition variables. Taking partnership status first, this had a limited impact on the frequency of drinking for men and women. Partnership status was not significant for the frequency of drinking at baseline for men, though there was a marginally significant finding for men not in a partnership (*p* < 0.10). This group of men, after controlling for wealth, education and smoking (*i.e.* Model 5 only), reported drinking more frequently at wave 0 in comparison to men who remained in a partnership. The interaction with wave shows that men who were not in a partnership at each wave experienced a steeper decline in frequency of drinking over time compared to men who remained in a partnership. For women, any baseline associations between frequency and partnership status found in Model 2 were non-significant when other socio-economic variables were included in the full model. However, as for men, in comparison to those who remained in a partnership, women not in a partnership at all waves experienced a more pronounced decline in drinking frequency over time, and the same was also found for women whose partnership ended during the observation period. Retirement also had a limited impact on the frequency of drinking and there were no consistent relationships between employment transitions and drinking frequency for men and women. In the full models for both genders there were no significant coefficients (*p* < 0.05), though for women being retired all the time or those who experienced an alternative employment transition marginally increased the frequency of drinking at wave 0 (*p* < 0.10).

In contrast to partnership and employment, self-rated health was more strongly associated with the frequency of drinking at the baseline and over time. The coefficients for both baseline and slope (*i.e.* the interaction terms between health and wave) were larger in comparison to the other transitions variables and were, mostly, significant. These coefficients were also consistent for men and women, though they were greater for the latter. At wave 0 respondents with poor self-rated health drank less frequently; as measured by the coefficients for those in continual poor and improving self-rated health as both groups had poor self-rated health at wave 0. The interaction with wave shows that those in continual poor self-rated health experienced a sharper decline in drinking frequency compared to those who remained in good health, and the same effect is observed for those whose health deteriorated between waves. For women, this latter group had a more pronounced decline in the frequency of drinking than those in permanent poor health.

The final group of variables included in the full model adjusted for socio-economic characteristics. These were significant for both men and women and illustrate that frequency of drinking in later life increased with higher wealth, levels of education, and for current and former smokers. For both men and women, and particularly for the latter, the largest coefficients were found for these variables, indicating the importance of resources and other health behaviours for patterns of alcohol consumption in later life.

### Weekly units consumed

Turning to the results for the analysis of the number of units of alcohol consumed in the last week (Tables 5 and 6), the findings were broadly similar to frequency though there were some important differences. In these tables, a negative coefficient for the baseline variables indicates that the category of interest drank less at wave 0 compared to the reference category; positive coefficients illustrate the opposite relationship. For the interactions between the lifecourse variables and wave, negative coefficients indicate that the category of interest was associated with a greater rate of change over time when compared with the reference category, *i.e.* the decline in weekly units consumed was accelerated.

**Table 5.** Growth curve model of units consumed in previous week (log): men

Variable	Model 1	Model 2	Model 3	Model 4	Model 5
Wave^1^	−0.077**	−0.075**	−0.071**	−0.069**	−0.066**
Age at wave 0	−0.023**	−0.024**	−0.022**	−0.023**	−0.021**
Partnership (Ref. In partnership all waves):					
Out of partnership all waves		0.287**			0.318**
Enters partnership between waves		0.485**			0.483**
Partnership ends between waves		0.009			−0.016
Partnership × wave:					
Out of partnership all waves		−0.008			−0.004
Enters partnership between waves		−0.027			−0.028
Partnership ends between waves		0.025			0.030*
Employment (Ref. Employed at all waves):					
Retired at all waves			−0.036		−0.072
Retires between waves			−0.131*		−0.137**
Other transitions			0.059		0.046
Employment × wave:					
Retired at all waves			−0.015		−0.015
Retires during study period			0.009		0.010
Other transitions			−0.029*		−0.026*
Self-rated health (Ref. Good health all waves):					
Poor health all waves				−0.079	0.039
Health deteriorates between waves				0.050	0.014
Health improves between waves				−0.145*	−0.147*
Self-rated health × wave:					
Poor health all waves				−0.025*	−0.023*
Health deteriorates between waves				−0.042**	−0.043**
Health improves between waves				−0.006	−0.007
Wealth (Ref. Bottom quintile):					
Second quintile					0.151*
Third quintile					0.059
Fourth quintile					0.105
Top quintile					0.215**
Education (Ref. No education):					
Compulsory education					−0.068
Post-compulsory education					−0.034
Degree or higher					0.173**
Smoking (Ref. Non-smoker):					
Used to smoke occasionally					−0.000
Used to smoke regularly					0.339**
Current smoker					0.377**
Constant	2.809**	2.753**	2.838**	2.804**	2.416**

*Notes*: English Longitudinal Study of Ageing participants in all three waves who are weekly drinkers in at least one wave; N = 1,774. 1. In Models 2–5 the coefficient for wave is for the reference group only for partnership (Model 2), employment (Model 3), health (Model 4) and for all transitions variables (Model 5). Ref.: reference category.

*Significance levels*: * *p* < 0.1, ** *p* < 0.05.

**Table 6.** Growth curve model of units consumed in previous week (log): women

Variable	Model 1	Model 2	Model 3	Model 4	Model 5
Wave^1^	−0.075**	−0.066**	−0.089**	−0.068**	−0.082**
Age at wave 0	−0.012**	−0.011**	−0.009**	−0.012**	−0.006**
Partnership (Ref. In partnership all waves):					
Out of partnership all waves		0.016			0.023
Enters partnership between waves		0.055			−0.004
Partnership ends between waves		−0.022			0.021
Partnership × wave:					
Out of partnership all waves		−0.012			−0.010
Enters partnership between waves		−0.014			−0.005
Partnership ends between waves		−0.033**			−0.030**
Employment (Ref. Employed at all waves):					
Retired at all waves			−0.184**		−0.198**
Retires between waves			−0.155**		−0.147**
Other transitions			−0.202**		−0.205**
Employment × wave:					
Retired at all waves			0.012		0.021
Retires during study period			0.019		0.023
Other transitions			0.016		0.023
Self-rated health (Ref. Good health all waves):					
Poor health all waves				−0.043	−0.010
Health deteriorates between waves				0.080	0.110*
Health improves between waves				−0.074	−0.044
Self-rated health × wave:					
Poor health all waves				−0.012	−0.011
Health deteriorates between waves				−0.035**	−0.037**
Health improves between waves				−0.010	−0.009
Wealth (Ref. Bottom quintile):					
Second quintile					−0.008
Third quintile					−0.051
Fourth quintile					0.142*
Top quintile					0.254**
Education (Ref. No education):					
Compulsory education					0.042
Post-compulsory education					0.090*
Degree or higher					0.313**
Smoking (Ref. Non-smoker):					
Used to smoke occasionally					0.198**
Used to smoke regularly					0.301**
Current smoker					0.378**
Constant	1.930**	1.909**	2.036**	1.932**	1.647**

*Notes*: English Longitudinal Study of Ageing participants in all three waves who are weekly drinkers in at least one wave; N = 1,879. 1. In Models 2–5 the coefficient for wave is for the reference group only for partnership (Model 2), employment (Model 3), health (Model 4) and for all transitions variables (Model 5). Ref.: reference category.

*Significance levels*: * *p* < 0.1, ** *p* < 0.05.

In Model 1 both age and wave variables were negative and significant, illustrating that the amount of alcohol that older adults consumed declined with age and over time. The coefficients for wave were similar for men and women, indicating a similar rate of decline over time by gender, though the coefficient for age at wave 0 was greater for men than women.

Turning to the lifecourse variables, for partnership we found differences by gender and a contrast to the results for the models of frequency. Taking men first, men not in a partnership in wave 0 (including those who subsequently re-entered a partnership by wave 5) were drinking more than men in a partnership at the baseline. These baseline coefficients for men not in a partnership were greater than coefficients for all other variables except for smoking, which demonstrates the importance of partnership status for men's baseline drinking. For women, partnership status had no significant association with baseline weekly alcohol consumption. However, women whose partnership ended during the study period experienced a steeper decline of weekly units over time compared to women who remained in a partnership and this trend replicates the same finding for frequency. It would appear that partnership was protective for men regarding how much they drank at the baseline, but not how often. While for women the end of a partnership led to a decline in both the frequency and quantity of alcohol consumption over time.

Retirement was not important for men's baseline drinking, which was similar to the results for the frequency model, though men who retired over the period of observation had a steeper decline in drinking than men who remained in employment. Retired women, those who retired between the waves and women who experienced another employment transition all drank less at wave 0 than employed women. For health, at wave 0 men with poor self-rated health whose health subsequently improved drank less, while women whose health deteriorated drank more, though these coefficients were only marginally significant (*p* < 0.10). Both men and women with deteriorating self-rated health reported a steeper decline in weekly consumption of alcohol over time compared to older adults who remained in good health. This suggests that worsening self-rated health was associated with a reduction in the amount of alcohol consumed over time.

The results for the socio-economic variables were similar to the model of frequency. However, unlike the frequency models in which there were differences between all categories, the differences by wealth quintile and education status for quantity were only found between older adults at the top and bottom of the wealth distribution and those with no or higher-level qualifications. For women, it was only the top two income groups that drank more than those in the bottom group. For men, there was a U-shape relationship; those in the second and top quintile reported more drinking. For education, both men and women with degrees drank more than those with no qualifications, and the same association was found for women with post-compulsory education. All female former and current smokers drank more in wave 0 than non-smokers, while for men this was restricted to current and former regular smokers.

## Discussion

The analysis of frequency and quantity of drinking in later life confirmed an overall decline in drinking behaviours in later life (Brennan *et al*. 2011; Breslow and Smothers 2004; Platt, Sloan and Costanzo 2010). We have found that this sample of people aged 45 and over experienced a decline in drinking over the ten years, and they also drank less often, which contrasts with cross-sectional data that show more frequent drinking in later life (Ng Fat and Fuller 2012). This suggests that while the current generation of older adults report drinking more often than younger drinkers, this behaviour is not necessarily associated with an increase in the frequency of drinking with age. Instead these longitudinal findings, when compared with recent cross-sectional data, indicate that there are cohort differences in the frequency of drinking and current younger cohorts have developed more time-focused drinking behaviours. The cohort of older adults considered in this analysis has experienced a particular constellation of economic and social changes which will shape their drinking behaviours. In particular, younger members (aged roughly between 45 and 65 in the late 1990s when data for wave 0 were collected), entered adulthood at the same time as per capita alcohol consumption increased in post-war UK (from the early 1960s onwards, peaking in the early 2000s). Moreover, they have also entered later life at the same time as economic prosperity for older people has improved relative to younger age groups (Belfield *et al*. 2014). Recent survey data that demonstrate continuation in older people's drinking practices while younger ages are drinking less may, therefore, reflect the relative prosperity of older and younger cohorts. Older adults have potentially more opportunities and more resources to facilitate the continuation of younger and mid-adult drinking practices into later life. Yet, as this analysis demonstrates, older people's drinking behaviours are mediated by the ageing process associated with overall declines in alcohol consumption and lifecourse transitions. The drinking behaviours observed in the current generation of older adults reflect a hybrid of established behaviours from younger and mid-adulthood; responses to an ageing body and associated lifecourse transitions; and, the prevailing social-economic context, which for the current generation of older people may facilitate the continuation of regular alcohol consumption.

Drinking is, for most people, a deeply social activity (Emslie, Hunt and Lyons 2012) and one that resonates with groups and individuals (Backett and Davison 1995); it is therefore reasonable to assume that lifecourse events in later life which bring about reorientations of older people's social interactions will impact on their drinking behaviours. However, the direction and magnitude of this impact has not been explored, which has been addressed in this analysis. The findings for health confirm that poor self-rated health was associated with drinking less often at the baseline and that those with poor or deteriorating health were more likely to reduce the number of times that they drank over time compared to those with good health (Balsa *et al*. 2008; Platt, Sloan and Costanzo 2010). Older adults whose self-rated health improved over the observation period initially drank less often when their health was poor, but did not reduce the frequency of drinking over time compared to those in good health. The findings for the impact of self-rated health on the number of units consumed were similar, yet overall the changes in self-rated health were more strongly associated with the frequency of drinking rather than with the quantity of alcohol that drinkers consumed. This could reflect fewer opportunities to drink among older adults with poor self-rated health. These findings suggest that changes in drinking behaviour associated with health occur because individuals are unwell and receive medical advice to reduce alcohol consumption (Shaper *et al.* 1988) or due to interactions with medication (Moore, Whiteman and Ward 2007). In addition, older adults with poor health may have fewer social opportunities to drink, and therefore drink less often.

Partnership transitions were also associated with changes in drinking behaviour. The results show that women cut back on alcohol consumption if they do not have a partner, while presence of a female partner moderates the amount that men drink. Cross-national research on married couples' drinking behaviours has found that wives influence their husbands to drink less and our analysis would confirm this (Selin, Holmila and Knibbe 2009). Yet for men, being in a partnership only influenced how much they drank, not how often. One possible explanation is that the moderation of how much partnered men drank occurred because consumption is shared, for example a bottle of wine (and wine was the favoured beverage among the ELSA respondents), while single men may be less likely to share consumption. Our findings demonstrate that for women the end of a partnership in later life leads to a significant decline in drinking. For women, partnership did not influence drinking at the baseline, though change in partnership status did intensify the decline in alcohol consumption. In particular, women whose partnership ended experienced the steepest decline in frequency and quantity of alcohol consumption compared to all other partnership status. The analysis is not able to distinguish between different partnership transitions and cannot shed light on the impact of bereavement rather than separation or divorce. Yet what it does reveal is that the presence of a partner and the social dynamics of relationships are important for drinking behaviours and these are different for older men and women. For example, the importance of loss of a partner for women's alcohol consumption may reflect the fact that more older women experience widowhood compared to men (Arber, Davidson and Ginn 2003). These findings for partnership contradict qualitative research that suggest being widowed is a causal factor for alcohol abuse (Wilson *et al*. 2013), but correspond with analysis of stress events which has shown that stress reduces the risk of women's alcohol misuse in later life (Sacco, Bucholz and Harrington 2014). This is an important area for further research to understand how and why partnership is an important, but different, determinant of both men's and women's drinking in later life, and how this might vary as the dynamics of relationships change in later life (Arber, Davidson and Ginn 2003).

For retirement, our analysis confirmed previous results that there is no definitive association between retirement and drinking behaviours (Brennan, Schutte and Moos 2010*b*; Kuerbis and Sacco 2012; Wang, Steier and Gallo 2014). One of the limitations of the employment transition variable is that it is difficult to summarise the complexity of retirement transitions, particularly for women, as nearly one-third of women in the sample experienced ‘other transitions’ or had missing data for this variable. However, despite this limitation, being in employment was found to be associated with greater weekly consumption for women. What is rather surprising is that retirement was more significant for women's drinking compared to men. For men, any relationship between drinking and retirement was explained by other socio-economic characteristics. The analysis therefore confirms the heterogeneity of retirement transitions and that for men, in particular, it is individual circumstances that are related to drinking, rather than the transition to retirement.

Analyses of baseline drinking confirmed that it was wealthier, better-educated men and women, as well as current and former smokers, who drank more and more often (Brennan, Schutte and Moos 2010*b*; Grittner *et al*. 2013). The importance of both economic capital (as measured by wealth) and cultural capital (as indicated by education) confirm that drinking was mediated by individual's social status. In particular, resources were strongly associated with the frequency of drinking. This suggests that material resources are important in enabling older people to drink on a daily basis, but are less important in influencing how much older people drink when they do consume alcohol. It would appear that the influence of socio-economic characteristics on drinking is not just a question of affordability, but that more affluent social groups cultivate daily drinking practices, while those with fewer resources limit the number of days on which they drink. Research to date has focused on the importance of social and cultural influences for younger people's drinking (van Wersch and Walker 2009); our analysis would suggest that this continues in later life.

## Limitations

There are a number of limitations to this study. First, the ELSA study is a household sample recruited from participants to the HSE, and this does not necessarily provide an appropriate sampling frame for identifying alcohol misuse. While there are individuals in the sample who consumed excessive quantities of alcohol (3 per cent of the sample, or 333 cases, in wave 0 reported heavy drinking, defined as drinking more than 50 units a week for men and 35 for women), the numbers were too small to provide detail on the risks of alcohol misuse in later life and our analysis has not considered these risks. Moreover, due to the number of different permutations we have had to simplify the lifecourse transition variables, and in particular have not distinguished between reasons for end of partnership.

## Conclusion

The analyses of lifecourse transitions and alcohol consumption confirm the importance of declining health in influencing how often and how much older adults drink. These results suggest that among older adults, those who drink more often have better health, and regular alcohol consumption in later life is indicative of good health. Yet the findings for lifecourse transitions relating to partnership and employment are less consistent and vary by gender. In particular, the differences between men's and women's partnership status and the amount that they drink suggest that the social context of alcohol consumption is gendered. Lifecourse transitions that impact on older people's social connectivity may lead to less drinking (in the case of health for men and women, and partnership and employment for women), but the results for partnership status for men hint at the possibility that some forms of social disconnection are associated with drinking more. Drinking in later life is just as much a social activity as it is at younger ages and one that older adults do because they enjoy a drink and derive some pleasure out of drinking with others. Our analysis has shown that as social relationships change in later life, these may impact on drinking behaviours, though the precise direction of this change varies according to the lifecourse event in question (health, employment or partnership) and by gender. These findings have implications for policies to support responsible drinking in later life, as they show that characteristics associated with improved wellbeing in later life (relating to resources and health) are also linked with drinking more and drinking more often. This highlights the importance of situating alcohol consumption within social and economic contexts and with reference to individual circumstances, as well as the possibility that policies to improve older people's wellbeing may have unintended consequences for alcohol consumption.

## NOTE

1 In the UK, one alcohol unit is measured as 10 millilitres (ml) or 8 grams of pure alcohol. This equals one 25 ml measure of whisky (alcohol by volume 40 per cent); one-third of a pint of beer (alcohol by volume ≈ 5 per cent) or half a standard (175 ml) glass of red wine (alcohol by volume 12 per cent).

## Appendix

**Table A1.** Distribution of variables

Variable	Men		Women	
	All respondents	Drinkers in at least one wave	All respondents	Drinkers in at least one wave
Mean age at wave 0 (SD)	61.0 (8.0)	60.5 (7.8)	61.8 (8.6)	60.5 (8.3)
	*Frequency (%)*			
Partnership:				
In partnership all waves	359 (17.5)	283 (16.9)	920 (34.2)	468 (29.6)
Out of partnership all waves	1,449 (70.8)	1,200 (71.5)	1,330 (49.4)	853 (53.9)
Enters partnership between waves	53 (2.6)	46 (2.7)	41 (1.5)	34 (2.2)
Partnership ends between waves	148 (7.2)	118 (7.0)	347 (12.9)	186 (11.8)
Other/missing	38 (1.9)	31 (1.9)	55 (2.0)	41 (2.6)
Employment:				
Employed at all waves	374 (18.3)	320 (19.1)	339 (12.6)	216 (13.7)
Retired at all waves	686 (33.5)	532 (31.7)	690 (25.6)	375 (23.7)
Retires between waves	701 (34.3)	589 (35.1)	868 (32.2)	540 (34.1)
Other/missing	286 (14.0)	237 (14.1)	796 (29.6)	451 (28.5)
Self-rated health:				
Good health all waves	1,215 (59.4)	1,047 (62.4)	1,537 (57.1)	1,019 (64.4)
Poor health all waves	316 (15.4)	232 (13.8)	432 (16.0)	173 (10.9)
Health deteriorates between waves	242 (11.8)	191 (11.4)	354 (13.2)	195 (12.3)
Health improves between waves	214 (10.5)	157 (9.4)	283 (10.5)	145 (9.2)
Other/missing	60 (2.9)	51 (3.0)	87 (3.2)	50 (3.2)
Wealth:^1^				
Bottom quintile	230 (11.2)	160 (9.5)	390 (14.5)	157 (9.9)
Second quintile	351 (17.2)	281 (16.8)	509 (18.9)	252 (15.9)
Third quintile	403 (19.7)	331 (19.7)	551 (20.5)	308 (19.5)
Fourth quintile	492 (24.0)	401 (23.9)	567 (21.1)	371 (23.5)
Top quintile	544 (26.6)	484 (28.8)	624 (23.2)	458 (29.0)
Missing	27 (1.3)	21 (1.3)	52 (1.9)	36 (2.4)
Education:				
No education	599 (29.3)	447 (26.6)	1,111 (41.3)	544 (34.4)
Compulsory education	577 (28.2)	479 (28.6)	889 (33.0)	546 (34.5)
Post-compulsory education	487 (23.8)	406 (24.2)	438 (16.3)	297 (18.8)
Degree or higher	384 (18.8)	346 (20.6)	254 (9.4)	195 (12.3)
Missing/not known	0	0	1	0
Smoking:				
Non-smoker	628 (30.7)	495 (29.5)	1227 (45.6)	689 (43.6)
Used to smoke occasionally	113 (5.5)	84 (5.0)	187 (6.9)	112 (7.0)
Used to smoke regularly	999 (48.6)	859 (51.2)	803 (29.8)	508 (32.1)
Current smoker	307 (15.0)	240 (14.3)	474 (17.6)	272 (17.2)
Missing	0	0	2 (0.1)	1 (0.1)

*Notes*: SD: standard deviation. 1. The distribution of wealth refers to wave 1.
